# A novel series of putative *Brugia malayi* histone demethylase inhibitors as potential anti-filarial drugs

**DOI:** 10.1371/journal.pntd.0010216

**Published:** 2022-03-16

**Authors:** Tamara Kreiss, Tyler Eck, Brittany Hart, Sreedhar Tummalapalli, David Rotella, John Siekierka

**Affiliations:** 1 Montclair State University and the Sokol Institute of Pharmaceutical Life Sciences, Montclair, New Jersey, United States of America; 2 Department of Microbiology, Biochemistry and Molecular Genetics. New Jersey Medical School, Newark, New Jersey, United States of America; Istituto Superiore di Sanità, ITALY

## Abstract

Filariasis, caused by a family of parasitic nematodes, affects millions of individuals throughout the tropics and is a major cause of acute and chronic morbidity. Current drugs are largely used in mass drug administration programs aimed at controlling the spread of disease by killing microfilariae, larval forms of the parasite responsible for transmission from humans to humans through insect vectors with limited efficacy against adult parasites. Although these drugs are effective, in some cases there are toxic liabilities. In case of loiasis which is caused by the parasitic eyeworm *Loa loa*, mass drug administration is contraindicative due to severe side effects of microfilariae killing, which can be life threatening. Our screening program and medicinal chemistry efforts have led to the identification of a novel series of compounds with potent killing activity against adult filarial parasites and minimal activity against microfilariae. A structural comparison search of our compounds demonstrated a close structural similarity to a recently described histone demethylase inhibitor, GSKJ1/4 which also exhibits selective adult parasite killing. We demonstrated a modification of histone methylation in *Brugia malayi* parasites treated with our compounds which might indicate that the mode of drug action is at the level of histone methylation. Our results indicate that targeting *B*. *malayi* and other filarial parasite demethylases may offer a novel approach for the development of a new class of macrofilaricidal therapeutics.

## Author summary

Parasitic diseases are responsible for tremendous suffering and morbidly throughout the world. There is a need for new drugs to treat parasitic disease. New approaches aimed at treating parasitic diseases depend upon an understanding of the critical functions required for parasite survival. One such disease is lymphatic filariasis (elephantiasis), a devastating parasitic disease initiated by the bite of a mosquito carrying infectious larvae. The larvae develop into adult parasites which sequester themselves in host lymphatics where they survive for several years. We have identified a novel series of compounds which appears to affect filarial parasite epigenetic regulatory mechanisms compromising parasite survival. These compounds provide both a pharmacological probe for the study of filarial parasite epigenetic mechanisms important for survival as well as providing a potential opportunity for anti-filarial drug development. In addition, these mechanisms are present in a variety of parasitic nematodes offering the opportunity for development of treatments for a wide variety of parasitic nematodes.

## Introduction

Filarial diseases such as onchocerciasis (River blindness), lymphatic filariasis (elephantiasis) and loiasis (*Loa loa* infection) are caused by a group of parasitic nematodes transmitted to humans through the bite of infected black flies, mosquitos and mango flies, respectively. These diseases result in significant morbidity along with economic and psychosocial impacts in endemic areas, disfiguring and incapacitating millions of individuals. Current mass drug administration (MDA) treatment options include ivermectin, albendazole and/or diethylcarbamazine which effectively eliminate microfilariae [1], the larval forms which are taken up by insect vectors, and are responsible for transmission of the disease, but not adult parasites. In one case, diethylcarbamazine treatment in *onchocerciasis* infected individuals can lead to serious and fatal side effects due to immunological responses to microfilaria killing [2]. There is an unmet medical need for new drugs which effectively target the adult parasites and limit responses to microfilariae killing, especially in *L*. *loa* co-infection. In addition, drugs targeting adult parasites may help in elimination programs by shortening the MDA.

In an effort to identify new anti-filarial drugs, in particular, drugs active against the adult parasites, we initiated phenotypic screening of several compound libraries, including the Medicines for Malaria Venture (MMV) Pathogen Box compound library, against adult and microfilariae of *Brugia malayi*. We identified two structurally related compounds, MMV658988 and MMV659004 (MSU-TSR-6-38), which exhibited inhibition of motility against adult *B*. *malayi* parasites and to a lesser extent against microfilariae. A number of analogs were then synthesized to explore physiochemical properties and potency of these MMV analogs against *Brugia malayi*, *Brugia pahangi* and *Litomosoides sigmodontis*. Four compounds, MSU-TSR-6-38, MSU-TSR-6-44, MSU-TSR-6-104 and MSU-TSR-7-006 were used as lead compounds for evaluation. One significant challenge associated with phenotypic screening in filarial parasites with compound libraries is identifying the molecular target (s) for the active compounds. To identify the putative parasite target (s) of the actives, we took an indirect approach whereby we performed a chemical structural similarity search between our active compounds and any potentially related compounds having a defined molecular target. Histone modifying enzymes are highly conserved in eukaryotes. DNA is wound up on a histone octamer core consisting of histones H2A, H2B, H3 and H4. Histones can be post translationally modified by a variety of mechanisms such as methylation and acetylation leading to changes in condensation of DNA, making regions inaccessible to transcription machinery, or promoting relaxation, allowing gene expression [3,4]. Methylation and acetylation are well studied post translational changes affecting histones. Lysine residues can be modified by methylation and can be either mono-, di- or trimethylated. Lysine methylation can either lead to activation or inactivation of gene transcription depending on the particular lysine residue methylated. The demethylase inhibitor GSK-J4, specifically targets the Jumonji C (JmjC) domain containing histone demethylases such as human UTX1 [5]. The JmjC domain is the catalytic domain responsible for demethylating histones by an oxidative mechanism requiring Fe(II) and alpha-ketoglutarate (αKG) as cofactors and is highly conserved in *B*. *malayi*. GSK-J4 has recently been reported to exhibit anti-parasitic activity against the parasitic trematode, *Schistosoma mansoni (S*. *mansoni)*, a parasitic nematode distinct from *B*. *malayi*, presumably through inhibition of demethylase activity, although a direct effect on parasite histone methylation has not been demonstrated [6]. Interestingly, treatment of parasites with our active compounds lead to modification of relevant methylated histones while treatment with GSK-J4 does not, as reported for S. mansoni [6]. In order to gain a better understanding of the mode of action of this class of compounds we performed a global RNA sequencing analysis of drug treated parasites. Our data provides evidence that the compounds affect histone modifying genes and downregulate muscle gene expression which affect contractile function. These results are reminiscent of those reported for GSK-J4 treated mice where muscle regeneration was impaired [7]. Data presented here indicate that *B*. *malayi* demethylases are potentially a novel epigenetic drug target in adult *B*. *malayi* with reduced activity and thus selectivity against microfilariae.

## Methods

### Worm treatment

All filarial parasites were obtained from the NIAID/NIH Filariasis Research Reagent Resource Center (FR3, Athens GA) *B*. *malayi* or TRS Laboratories (Athens, GA) *B*. *pahangi* or *L*. *sigmodontis*. Adult parasites, harvested from infected jirds, were plated in 24-well plates with 2 mL of Advanced RPMI 1640 medium (Invitrogen) supplemented with 25mM HEPES, 2 mM L-Glutamine (Invitrogen), 100 U/mL Penicillin (Invitrogen), 100 g/mL Streptomycin (Invitrogen), 2.5 g/mL Amphotericin B solution (Invitrogen), and 5% inactivated fetal bovine serum and placed in a 37°C humidified incubator with 5% CO_2_. The worms (1 worm per well, 6 wells per treatment group) were transferred into a new plate containing fresh media and drug (1 μM), or vehicle control (0.1% DMSO) every 48 hours. Our experiments concluded on day 5. Motility of the adult worms and microfilariae (~100 microfilaria per well with 8 replicate wells per treatment group) was given a score by sight from 0 to 4 with 4, rapid movement and largely coiled; 3, moderate movement and uncoiled; 2, slow movement and uncoiled; 1, twitching movement and uncoiled; 0, no motility (dead). Viability was also assessed colorimetrically using 3-(4,5-dimethylthiazol-2-yl)-2,5-diphenyltetrazolium bromide (MTT) following a 48-hour incubation. The assay was performed as described previously [8]. All experiments were repeated 2–3 times.

### RNA sequencing

*Brugia malayi* male worms treated with the compounds MSU-TSR-6-38 and MSU-TSR-6-44 in comparison to a DMSO treatment control group were used for transcriptomic RNA analysis (~30 worms per group). The protocol was adapted from the Filarial Research Reagent Resource Center (FR3), GA. Male parasites were treated with compounds at 100 nM in media for 4 hours and briefly washed in sterile 1x PBS. The parasites were transferred to 2 mL round bottom tubes (LoBind Eppendorf) containing 100 μL lysis solution RNAqueous Micro Kit (AM1931) and a 3.2 mm stainless steel bead (BioSpec Cat. No. 11079132ss) at 4°C before being flash frozen in liquid nitrogen. Parasites underwent three freeze-thaw cycles before being lysed for 45 minutes on a VortexGenie with a horizontal tube adapter. After lysis, 50 μL of EtOH (96% ACS grade) was added to each 100 μL of worm lysis solution. The parasite lysis solutions were spun down at max speed for 10 minutes and the RNAqueous Micro Kit protocol was followed. In brief, clear supernatant was loaded into the Micro Filter Cartridge Assembly unit and the supernatant was spun down. Samples were washed with Wash buffer 1 once and Wash buffer 2/3 twice with centrifugation between each step. Centrifugation never exceeded 15,000 x g as the filter potentially could get compromised. A last spin was performed for 5 minutes into an empty tube to ensure all solutions were removed. Cartridges were placed into a new clean collection tube and eluted twice with 10 μL pre-warmed elution buffer. Samples were stored at -80°C. Samples were shipped on dry ice to GeneWiz (Piscataway, NJ) for quality control and RNA sequencing on an Illumina HiSeq2500, 1x50bp single-read (SR) configuration in High Output mode (V4 chemistry) and differential gene expression analysis. Briefly, Fastq files for 4 samples were retrieved, sequence reads trimmed to remove low quality bases at ends, sequence reads mapped to the *B*. *malayi* WS254 reference genome using CLC Genomics Workbench v. 10.0.1, total gene hit counts and RPKM values for genes calculated, and gene expression compared between single samples using Kal’s Z-test. All samples contained high quality data. Detected *B*. *malayi* transcripts were received in WormBaseID format along with corresponding data and statistics. To obtain gene ontologies and molecular functions of affected genes, WormBaseID’s were converted to Uniprot accession numbers before using the Panther Classification Gene Analysis web tool [9,10].

### Histone extraction and purification

*Brugia malayi* female worms were treated (30 worms in 1 well, 8 mL. Repeated 3 times) in the absence and presence of the active drugs MSU-TSR-6-104, MSU-TSR-7-006, GSK-J4 and Emodepside at 1 μM for 18 hrs. Parasites were briefly rinsed in 1x PBS and in 2 mL round bottom tubes (LoBind Eppendorf) containing about 400 μL extraction buffer Histone Purification Kit No. 40025 (Active Motif) 1 x Halt Protease Inhibitor (Pierce), PMSF and a 3.2 mm stainless steel bead (BioSpec Cat. No. 11079132ss). Worms were lysed for 45 minutes on a Vortex Genie with a horizontal tube adapter at 4°C. The lysis solution was spun down at 20,000 x g for 15 minutes. Supernatant was collected and neutralized with 5X Neutralization buffer (Kit 40025, Active Motif). Histones were purified by a small gravity-flow column, with resin that was prepared according to the manufacturer’s protocol. Total purified parasite histones H2A/H2B and H3/H4 were concentrated in a Spin-X UF 6 mL Centrifugal Concentrator, 5,000 MWCO (Corning).

### SDS-PAGE and Western Blot analysis

Purified histone samples were analyzed using SDS gel electrophoresis (SDS-PAGE). Samples were prepared in NuPAGE SDS Sample Buffer (Invitrogen) containing NuPAGE Reducing Agent (Invitrogen) and incubated at 90°C for 5 minutes prior to loading them on a NuPAGE 12% Bis-Tris gel (Invitrogen). Gels were run at a constant voltage (200 V) for 40 minutes in MOPS-SDS running buffer (Invitrogen) using Novex Sharp Pre-Stained Protein Standards (Invitrogen). Protein was transferred from SDS-PAGE gels onto a polyvinylidenefluoride (PVDF) membrane using NuPAGE Transfer Buffer (Invitrogen) containing 10% methanol and 0.25% SDS using a TE77XP Semi-Dry Blotter (Hoefer, Holliston, MA) for 1 hour at 54 mA per blot. After transfer, membranes were placed in TrueBlack (Biotium) blocking buffer for 45 minutes. Blots were either incubated with Tri-Methyl-Histone H3 (Lys27) (C36B11) Rabbit mAb #9733 (Cell Signaling) 1:1000, Ubiquityl-Histone H2B (Lys120) (D11) XP Rabbit mAb #5546 (Cell signaling) 1:1000 or Tri-Methyl-Histone H3 (Lys4) Antibody #9727 Rabbit mAb (Cell Signaling) 1:1000 were used for primary antibodies in TrueBlack antibody diluent (Biotium) with 0.01% sodium azide for 1 hour. Membranes were then washed 3 times for 5 minutes with TBS-T. The secondary antibodies used were 1:10,000 IRDye 800CW Goat anti-Rabbit IgG Secondary Antibody 1: 10,000 (LI-COR) and IRDye 680RD Goat anti-Rabbit IgG Secondary Antibody 1:10,000 (LI-COR). Membranes were then washed 3 times for 5 minutes with TBS-T and allowed to dry. The blots were imaged on an Odyssey CLx (LI-COR).

### Enzymatic assay

The truncated BmUTX-1 encoding region of the gene (Bm3036: amino acids 579–1090) was synthesized (Genscript, Piscataway NJ) and inserted into a Gateway pDONOR21 vector (Invitrogen). An LR clonase reaction was performed according to the manufacturer’s (Invitrogen) protocol to insert the gene into a pDEST 15 expression vector (Invitrogen) vector, which allows the protein to be expressed with an N-terminal GST affinity tag. The GST-Bm3036 (aa 579–1090) construct was expressed in BL21 DE3 Star cells by inoculating a 250 mL culture containing 100 μg/mL carbenicillin, and incubating overnight at 37°C with shaking at 225 RPM. Cells were pelleted at 10,000 x g for 10 minutes and lysed with ice-cold B-PER extraction reagent containing 1X protease inhibitor (Pierce). Following incubation for 10 minutes, the lysate was pelleted at 15,000 x g for 15 minutes and the supernatant was collected. GST-Bm3036 (aa 579–1090) purified by antibody pulldown using a Mouse anti-GST antibody and the Dynabeads Protein G Immunoprecipitation Kit (Invitrogen). In brief, 100 μL of Dynabeads were washed and then incubated with 0.25 μg/ μL of the Mouse anti-GST antibody at RT for 10 minutes. Lysate was added in 2 mL increments, and incubated for 5 minutes at RT. Beads were washed 3 times before performing two elution steps of 50–100 μL.

The activity of recombinant GST-Bm3036 (aa 579–1090) was investigated using 10 μg of purified Bovine core histones as the substrate. Our demethylation assay buffer conditions were as follows: 50 mM HEPES (pH = 7.6), 150 mM NaCl, 2 mM ascorbic acid, 50 μM Fe(SO_4_), and 20 mM α-ketoglutarate. Following addition of Bm3036, the reaction was incubated overnight at 37°C. The reaction was evaluated by western blot analysis as described above, using the Tri-Methyl-Histone H3 (Lys27) (C36B11) Rabbit mAb #9733 (Cell Signaling) 1:1000 antibody. Blots were imaged on an Odyssey CLx (LI-COR).

### Protein/Structural analysis

NCBI’s blastp tool (https://blast.ncbi.nlm.nih.gov/Blast.cgi) was used to identify similar protein sequences to Bm3036 in both nematodes and humans. To further this analysis, the WormBase translated sequence of Bm3036 was run through InterPro’s domain search tool (https://www.ebi.ac.uk/interpro/). Followed by blastp against similar domains in the human homologs and Bm3036, and a dot plot was formed. Sequence alignments were performed on Clustal Omega (https://www.ebi.ac.uk/Tools/msa/clustalo/).

### Statistical analysis

Statistical significance in the MTT assay was determined for multiple treatment groups by One-Way ANOVA. All groups tested were sufficiently normal (Shapiro-Wilkes *p* > 0.1) and homogeneity of variances (Levene’s test *p* = 0.476) was observed. Following a significant effect in the omnibus test, post-hoc pairwise t-tests were conducted between each compound treatment group against the 0.1% DMSO control (six tests). Alpha was corrected using the Bonferroni correction (α = 0.05 / 6 = 0.0083).

## Results

**Fig 1:**
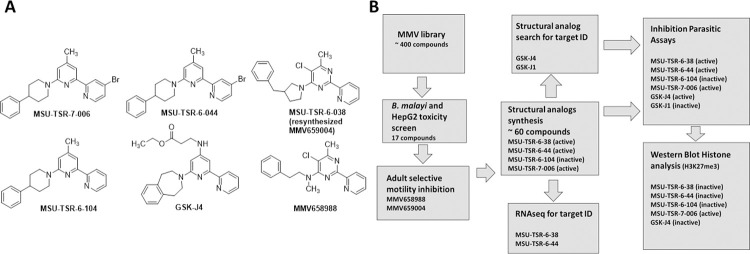
(A) Compounds identified, synthesized and tested for inhibition of parasite motility. (B) Flow diagram illustrating screening, synthesis and evaluation of compounds.

**Fig 2:**
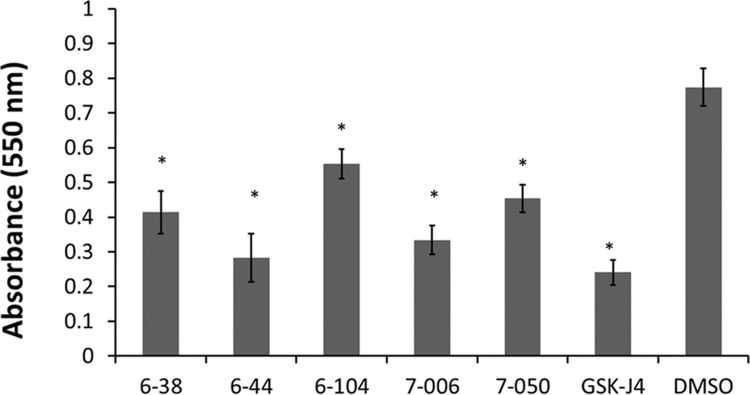
Effects of GSK-J4 and demethylase analogs on adult B. malayi viability. Female adult worms were cultured with 0.1% DMSO or 1 μM of compounds 6–38, 6–44, 6–104, 7–006, or GSK-J4 for 48 hours before evaluation by MTT assay. At least seven worms were used in each treatment group. Bars represent mean absorbance and error bars show SEM. Statistical significance was determined by ANOVA and post-hoc pairwise t-tests and are indicated by asterisks if p < 0.0083 (Bonferroni correction).

In an initial screen of 400 drugs in the Medicine for Malaria Venture (MMV) pathogen box, we identified 50 compounds that inhibited *B*. *malayi* worm motility. Of these, 17 were non-toxic in a HepG2 toxicity assay at 10 μM and after evaluation of their pharmaceutical properties (e.g. metabolic stability, water solubility) and structure, two compounds were selected, MMV658988 and MMV659004 (Fig 1), for further investigation. We initiated a medicinal chemistry effort to improve physiochemical properties and potency and consequently generated approximately 60 analogs, which were evaluated for their ability to reduce the motility against *B*. *malayi*, *B*. *pahangi* and *L*. *sigmodontis* adult worms (male and female) and microfilariae. Three compounds emerged as our top active compounds: MSU-TSR-6-38, MSU-TSR-6-44, and MSU-TSR-7-006. MSU-TSR-7-004 is an inactive analog of MSU-TSR-7-006 (Fig 1) These compounds exhibited potent inhibition of adult parasite motility at 1 μM (Table 1) and loss of viability as assessed by MTT assay (Fig 2). Interestingly, microfilariae were significantly less affected by these compounds then adults (Table 1). A structural similarity search against the pharmacophore, 4,6-dichloro-2-(2-pyridinyl)pyrimidine, a precursor for the synthesis of one of our candidates, MSU-TSR-6-044 (Fig 1), revealed close structural similarity to GSK-J4 (GlaxoSmithKline), an inhibitor of human histone demethylases JMJD3 and UTX-1. GSK-J1/J4 has activity in vitro and in vivo against various types of cancers and is also being investigated as an anti-inflammatory agent [3,5]. A comparison of the binding model for GSK-J1 with the human demethylase JMJD3 and the structure of MSU-TSR-6-044, suggests that the pyridine nitrogen atoms in MSU compounds can coordinate with the key active site metal in a manner similar to GSK-J1 and interact with the enzyme in a homologous manner. Our structure-activity observations associated with MSU-TSR-6-44 support this binding hypothesis.

**Table 1:** Percent parasite motility by various synthetic inhibitors and GSK-J4. Parasites were treated with indicated compounds at 1 μM for 48 hours. All experiments were repeated at least three times n = 3. Except for MSU-TSR-6-104 which is a structurally inactive analog and MSU-TSR-6-38, all compounds are selective towards adult parasitic stages. The compounds are also active against the closely related *B*. *pahangi* and the rodent nematode *L*. *sigmodontis*. All reported percent inhibition are within a ≤ 10% standard error. All compounds are inactive against *C*. *elegans* at 100 μM.

Compound	*B*. *malayi male*	*B*. *malayi female*	*B*. *malayi microfilaria*	*B*. *pahangi female*	*L*. *sigmodontis female*
MSU-TSR-6-38	n/a	12	31	12	0
MSU-TSR-6-44	6	12	62	0	0
MSU-TSR-6-104	87	94	100	94	100
MSU-TSR-7-006	6	50	75	25	25
GSK-J4	12	0	75	0	0

**Fig 3:**
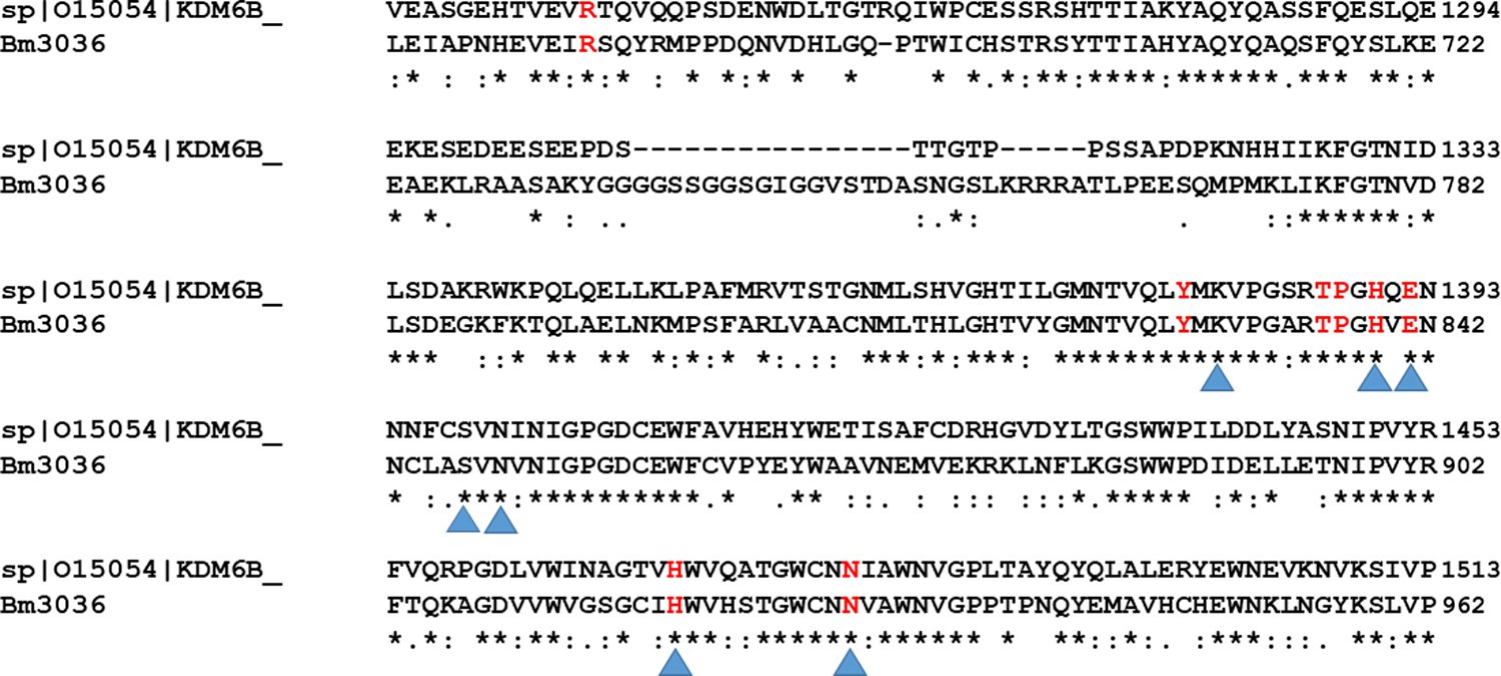
Residues in red represent contact sites for GSKJ1. Triangles represent contact sites for N-Oxalylglycine (NOG).

We performed a Clustal Omega alignment between human JMJD3 (Uniprot ID No. O15054), for which there is a crystal structure, and *B*. *malayi*, BmUTX1 (Bm3036), histone demethylase. A comparison of the catalytic JmjC C-terminal domains, indicates a high degree of homology between human and *B*. *malayi* demethylases (Fig 3). The highlighted amino acids indicate the specific interaction sites for the GSK-J1 inhibitor to JMJD3/UTX1 as well as the demethylase inhibitor N-oxalylglycine (NOG). These sites are conserved in *B*. *malayi* UTX-1, supporting our hypothesis that the target of our MSU anti-filarial compounds is a filarial histone demethylase(s).

**Fig 4:**
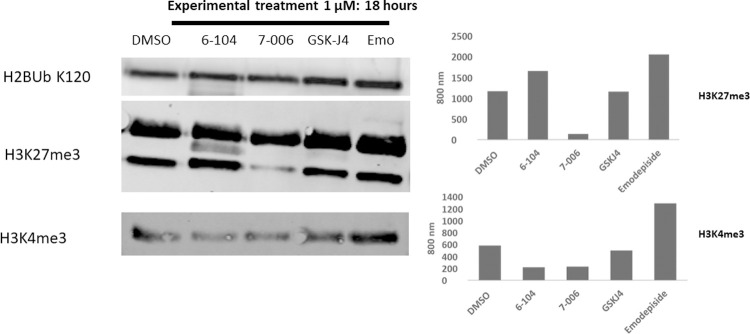
Left: B. malayi female western blot, treated with indicated compounds and vehicle control for 18 hours. Histones were extracted and purified and run on a 12% Bis-Tris gel. H2B ubiquitination on Lys120 was used as a loading control as it has been found to be constant regardless of experimental treatment in previous experiments. 6–104, an inactive structural analog was used as a negative control. 6–104, GSK-J4 and Emodepside do not appear to inhibit H3K27 demethylation. Right: Graphical representation of the western blot by normalization of H3K27me3 (top right) and H3K4me3 (bottom right) to loading control. Image: Odyssey CxL Image Studio.

Histone H3 is highly conserved between human and *B*. *malayi* (Uniprot identifiers Bm4536, Bm4100, and Bm3425) which allowed us to investigate methylation status in the parasite, using commercially available antibodies, upon inhibitor treatment. We chose to use MSU-TSR-7-006 for this analysis since we had an inactive structural analogue, MSU-TSR-6-104, available as a control compound (Fig 1) We found that MSU-TSR-7-006 treatment significantly altered the methylation status of H3K27me3 (Fig 4). The western blot shows two bands; an upper band with an unchanged methylation status, likely due to H3 phosphorylated on serine 28 previously shown to prevent demethylation [11], and a lower band with a significant attenuation in demethylation. Ubiquitinated histone H2B was used as a loading control as it has been found to be constant regardless of experimental treatment in previous experiments. MSU-TSR-6-104, GSK-J4 and the anti-helminthic drug Emodepside [12] did not alter H3K27 methylation status. Interestingly, the human histone demethylase inhibitor, GSK-J4, a pro-drug of GSK-J1, did not alter the methylation status of H3K27me3 (S1 Fig Demethylase Inhibition Assay) although exhibiting potent inhibition of parasite motility (Table 1). A similar observation was made with the anti-schistosomal activity of GSK-J4 where no histone modifications were observed upon drug treatment of parasites [6]. Our findings are the first to demonstrate a change in histone methylation in *B*. *malayi* with our putative histone demethylase inhibitors.

**Fig 5:**
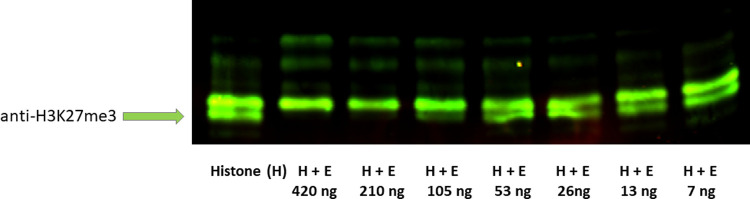
Recombinant B. malayi UTX-1 (E) serial diluted incubated with total bovine histone (H) constant. Recombinant B. malayi UTX-1 activity assay. Purified histones (600 ng) were used for each reaction with varying amounts of enzyme (420–7 ng). An anti-H3K27me3 was used to detect the methylation status after an overnight incubation. BmUTX-1 demethylates H3K27me3.

We further sought to confirm that the closest human UTX-1 orthologue in *B*. *malayi*, BmUTX-1, was capable of demethylating H3K27me3 and if our active inhibitors inhibited this activity. We expressed and purified a truncated, recombinant (AA 579–1090) protein encoded by BmUTX-1, and monitored histone demethylation of bovine histone extracts. We demonstrated that BmUTX-1 exhibits demethylation activity towards H3K27me3 (Fig 5), however attempts to inhibit BmUTX-1 demethylase activity with our active compounds or GSK-J4 were not successful. One reason for this may be due to the fact that *B*. *malayi* has a number of JmjC domain histone demethylases; Bm9277, Bm10151, Bm9447, Bm5385, Bma-jmjc-1, Bma-rbr-2, Bma-jmjd-4, Bma-jhdm-1, Bma-jmjd-2, Bma-psr-1, and Bma-jmjd-5 [www.wormbase.org.release 15. 2019]. The exact target for our inhibitors, therefore, would require a comprehensive evaluation of all JmjC domain containing proteins present in *B*. *malayi*. Adding to the complexity of inhibitory mechanism, histone demethylases are usually part of larger, multi-protein, regulatory networks that might modify activity and promote inhibition with our compounds and GSK-J4.

### Transcriptome analysis

**Fig 6:**
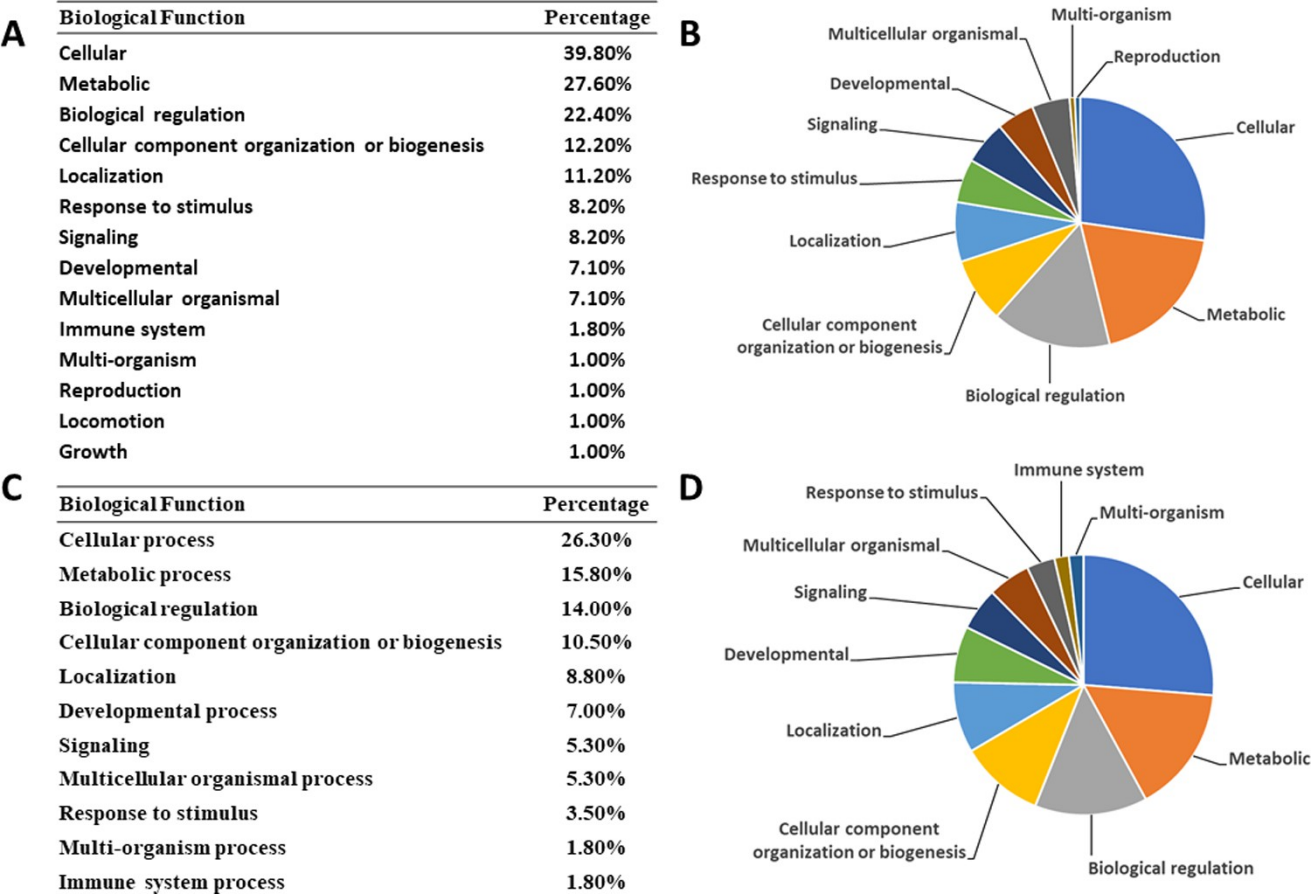
Biological functions affected by treatment of B. malayi males with 100 nM of MSU-TSR-6-44 (A and B) or MSU-TSR-6-38 (C and D). Gene classification was conducted with the Panther Classification System Gene Analysis tool, using the B. malayi Genome as a reference. (Mi H et. al. 2018; Mi H et. al. 2019).

Phenotypic screening of small molecules has the advantage of providing a desired readout but often comes with a significant trade off as the target(s) is unknown. While transcriptomic analysis does not directly allow us to assess the mode of inhibition, it does provide a landscape of which pathways in the presence of drugs are perturbed, when compared to the vehicle control. Since adult parasites are more sensitive towards this series of compounds, we were interested in obtaining RNA from the adult stage in the absence of embryonic and larval stages. *Brugia malayi* males give the advantage of providing a homogenous life stage without the need to manipulate female parasites. RNA sequencing was performed on *B*. *malayi* male worms treated with 100 nM of MSU-TSR-6-38 or MSU-TSR-6-44 to identify genes that were differentially expressed from a 0.1% DMSO vehicle control. The drug treatment at 100 nM for 4 hours did not have an effect on viability on the worms which is desirable as we did not want to turn on stress pathways that would mask any drug related responses. A total of 158 genes were found to be differentially expressed in the MSU-TSR-6-44 treatment by a factor of at least 2-fold—13 of which were found to be upregulated, while 145 were downregulated (S1 Data MSU-TSR-44). The less potent MSU-TSR-6-38 caused 13 genes to be upregulated and 56 to be downregulated, totaling 69 impacted genes (S2 Data MSU-TSR-6-38). Interestingly, 61 genes were found to be differentially expressed in both treatments, however, only 33 genes from the MSU-TSR-6-38 treatment were classifiable using the Panther Gene Analysis web tool [9,10]. Classification of these consensus genes show that cellular processes, metabolic processes, biological regulation, localization and development were predominantly affected (Fig 6).

Panther GO-Slim Biological Process classification of genes observed in both datasets revealed transcriptional regulation (RNA binders and RNA polymerases), the ubiquitin-proteasome pathway (E3 ubiquitin ligases, ubiquitin thioesterases, and ubiquitin hydrolases) as well as structural and contractile muscle functions (myosin, actin filament binding, and kinesin-like proteins) to be downregulated by compound treatments (S3 Data RNASeq Consensus Genes). This suggests that a muscle function is heavily impacted by these compounds. Additionally, further analysis of the more potent compound MSU-TSR-6-44 revealed histone modifiers (histone lysine N-methyltransferases and histone acetyltransferases) were downregulated. Using the WormBaseID numbers, these genes appear to be orthologs to the *C*. *elegans* genes set-2, set-12 and lys-12 (WBGene00235103, WBGene00222668, and WBGene00230743, respectively), which are suggested to control H3K4 methylation statuses as well as H3K9 and H3K27 acetylation [13,14]. A complete list of classifiable genes can be viewed in the supplement (S1–S3 Data files). All differential transcripts were analyzed using string-db.org multiple protein sequence analyzer. C. elegans was chosen as the closest model organism to create a network of potential protein interactions within the transcripts. Two major interacting clusters showed chromatin modifying genes as well as muscle specific genes that are important for contractile functions PPI enrichment p-value: < 1.0e-16. RNA sequencing analysis in the presence and absence of drug supports our hypothesis that histone demethylases are targeted, since muscle related genes that are important for physiological contractile function, were downregulated [5].

## Discussion

A phenotypic screen assessing inhibition of adult female *B*. *malayi* motility was performed using the MMV Pathogen Box compound library. Two actives were identified, MMV658988 and MMV659004. We synthesized several analogs of these compounds to improve potency, selectivity against adult parasites and drug like properties. These analogs were tested against *B*. *malayi*, *B*. *pahangi*, and *L*. *sigmodontis*. We found that these compounds show potent inhibitory activity against adult parasites with some selectivity over microfilaria (Table 1). A structural similarity search of our top two compounds, MSU-TSR-6-38 and MSU-TSR-6-44, revealed similarities to a JmjC demethylase inhibitor, GSK-J4. We synthesized GSK-J4 and determined that, like our compounds, it also has potent inhibitory activity against *B*. *malayi*. These observations indicated that a *B*. *malayi* demethylase may be the target for these compounds.

A structural alignment between human JMJD3 (Uniprot ID No. O15054) and the *B*. *malayi* ortholog, BmUTX-1, JmjC C-terminal domain, revealed a high degree of homology with high conservation of compound interaction sites further attesting to the target being a histone demethylase.

We evaluated the effects of the compounds MSU-TSR-6-38 and MSU-TSR-6-44 on the adult worms using RNA sequencing technology (RNAseq) to provide further information regarding the target for our active compounds as well as providing insight into the mechanistic consequences of inhibiting *B*. *malayi* demethylases. Our RNAseq study revealed three groups of related genes that were impacted by the more potent MSU-TSR-6-44 treatment (histone modifiers: histone lysine n-methyltransferases and histone acetyltransferases; muscle function: myosin light and heavy chains, kinesin-like proteins, and actin filament binding; and the ubiquitin-proteasome pathway: E3 ligases, ubiquitin thioesterases, and ubiquitin hydrolases [15]. In relation to the *C*. *elegans* ortholog, some impacted muscle genes include unc-104 striated muscle contractions, unc-15 skeletal muscle myosin thick filament assembly, ehbp-1 locomotion and striated muscle myosin thick filament assembly, and pat-12 which ensures muscle stability and muscle connection to the external cuticle. No histone demethylase transcripts were identified by the Panther GO-Slim Biological Process classification for these treatments. Although, epigenetic regulation was clearly impacted by the disruption of histone methyltransferases, histone acetylases, and the ubiquitin-proteasome pathway. The three identified histone modifiers, set-2, set-12, and lsy-12 are suggested to influence H3K4 methylation statuses as well as H3K9 and H3K27 acetylation [13,14]. Ubiquitination also is considered to be a layer of epigenetic regulation, since histones and their modifiers can be tagged with ubiquitin for degradation or as part of the epigenetic code [15,16]. Studies have also shown that E3 ligases can indirectly regulate the epigenetic code by targeting histone demethylases for degradation, as is observed between the KDM4A demethylase and SFCFBXO22 E3 ligase [16]. This muscle contraction-epigenetic regulation connection is consistent with other studies of the GSK inhibitors, as GSK-J4 which is the pro-drug of GSK-J1 demonstrated that the epigenetic inhibitor impaired muscle regeneration in mice [7]. The supposed targets of these GSK compounds, JMJD3 and UTX, both favor an active transcriptional state of muscle specific genes which can explain the downregulation of muscle related genes when these enzymes are inhibited [7]. Taken together, these results suggest that our GSK structural analogs may impact muscle contractions and epigenetic regulation in a similar manner to that of the GSK-J1/4 inhibitors.

A significant hurdle in identifying drug targets using phenotypic screens in *B*. *malayi* and other parasitic nematodes is the lack of genetic tools to verify targets. To circumvent this, we have evaluated our inhibitors against *C*. *elegans* in order to utilize the genetic tools available with this organism for drug target identification. We did not observe any effect of compounds against *C*. *elegans* regarding motility and viability. The lack of activity against *C*. *elegans* could be due to a number of issues. A high throughput screen of over 1000 drug like molecules exhibited problems with drug uptake in over 90% of *C*. *elegans* tested [17]. While a similar screen has not to our knowledge been conducted with *B*. *malayi*, it is possible that the cuticle composition of the soil nematode *C*. *elegans* is much more rigid and less permeable to xenobiotics due to its environmental niche, soil, when compared to long lived (10–15 years) *B*. *malayi* which resides in the sterile environment of the host lymphatics. *C*. *elegans* devotes a number of genes to deal with detoxification and drug metabolism expressing eighty cytochrome p450 (cyp450) enzymes, compared to *B*. *malayi* having five cyp450s (Wormbase). Lastly, given the genetic disparity between *C*. *elegans* and *B*. *malayi*, regulation and dependencies upon histone methylation processes may differ significantly.

One of the more striking observations in our studies is that our active compounds, as well as GSK-J4, are more selective towards adult parasites than microfilaria. While further studies are necessary to provide insights into this selectivity, we have found that RNA transcript levels of BmUTX-1 are much higher at the adult life stage compared to the microfilaria stages (http://www.wormbase.org, release WS277, date 08/2020.) The nine other JmjC domain containing genes did not follow this same trend (S4 Data Histone demethylase Expression Across Lifestages). However, RNA expression levels are often not predictive of total protein levels and it also does not allow us to determine the relevance of these genes in each individual life stage. An additional explanation could also be that larval growth is arrested at the L1 stage and remains relatively quiescent until the intermediary mosquito vector ingests the larvae [18]. At the L1 stage, larvae in growth arrest are less dependent on the epigenetic machinery, unlike adult worms that continue to molt and proliferate. One such marker for growth arrest is an increase in hypermethylated 5-methylcytosine [18] in DNA which warrants assessment in the studies presented here.

Lastly, it is worthwhile mentioning that the target BmUTX-1 is conserved amongst the major filarial human pathogens *Wuchereria bancrofti*, *Brugia tumori*, *Loa loa*, and *Onchocerca volvulus* with 90% or more sequence homology. In addition, the target is conserved in other parasitic nematode pathogens that affect human and animals, these include *Elaeophora elaphi*, *Thelazia callipaeda*, *Ascaris suum*, *Dracunculus medinensis*, *Enterobius vermicularis*, and *Toxocara canis*, potentially the use of our compounds as novel broad spectrum antiparasitic agents. It has been recently reported that GSK-J4 exhibits anti-parasitic activity against *Schistosoma mansoni* [6]. Schistosomes are trematodes who’s life cycle is quite distinct from *B*. *malayi*, involving freshwater snails as a vector. Treatment of *S*. *mansoni* parasites in vitro with GSK-J4 had a significant effect on parasite viability, motility and egg oviposition [6]. However, western blot analysis failed to reveal any modulation of the levels of H3K27me3 in drug treated worms. Similarly, we have observed significant anti-parasitic activity against B. *malayi* with GSK-J4, but as is the case for *S*. *mansoni*, have been unable to demonstrate modulation of H3K27me3 levels with this compound. This contrasts with our inhibitors which have a demonstrable effect on histone methylation status.

In summary, we have identified two novel histone demethylase inhibitors, MSU-TSR-6-38 and MSU-TSR-6-44 with potent activity against *B*. *malayi*. In addition, we have demonstrated that these compounds, along with GSK-J4, while potent of adult parasite motility, exhibit much less activity against microfilariae. This important property can be exploited for use in situations where killing of microfilariae leads to severe toxicity and mortality as in the case of loiasis where drugs such as ivermectin are contraindicated leaving few if any treatment options. The compounds described here are currently planned to be evaluated *in vivo* in a *B*. *malayi* infected gerbil model of filariasis.

## Supporting information

S1 Data MSU-TSR-6-44 Dataset: Panther GO-classification of differentially expressed genes in B. malayi adult males following MSU-TSR-6-44 treatment compared to DMSO control. A 2-fold cutoff was used to obtain the dataset, and all genes are accompanied by Uniprot ID, WormBase ID and WormBase descriptions.
(XLSX)

S2 Data MSU-TSR-6-38 Dataset: Panther GO-classification of differentially expressed genes in B. malayi adult males following MSU-TSR-6-38 treatment compared to DMSO control. A 2-fold cutoff was used to obtain the dataset, and all genes are accompanied by Uniprot ID, WormBase ID and WormBase descriptions.
(XLSX)

S3 Data RNAseq Consensus Genes Dataset: Panther GO-classification of differentially expressed genes that are represented in both the MSU-TSR-44 and MSU-TSR-38 treatment groups. A 2-fold cutoff was used to obtain the dataset, and all genes are accompanied by Uniprot ID, WormBase ID and WormBase descriptions.
(XLSX)

S4 Data Histone demethylase Expression Across Lifestages: Transcript expression provided for predicted histone demethylases are plotted against B. malayi lifestages. All values were taken from Wormbase. Tabs are separated as all predicted demethylases, and by jmjC-domain containing or lysin-specific demethylases.
(XLSX)

S1 Fig BmUTX-1 Inhibition Assay: MSU compound activity was tested against purified BmUTX-1 using bovine histones as a substrate. Tested compounds include MSU-TSR-038, 044, 104, 7–006, and GSK-J1. Based on the DMSO control lane, none of the compounds appeared to inhibit the ability for BmUTX-1 to demethylate the H3K27me3 mark.
(TIF)
