# Supplementary figures and images for: A novel series of putative Brugia malayi histone demethylase inhibitors as potential anti-filarial drugs
Source: PLoS Negl Trop Dis. 2022 Mar 16;16(3):e0010216. doi: 10.1371/journal.pntd.0010216 (PMC8926182; doi:10.1371/journal.pntd.0010216)

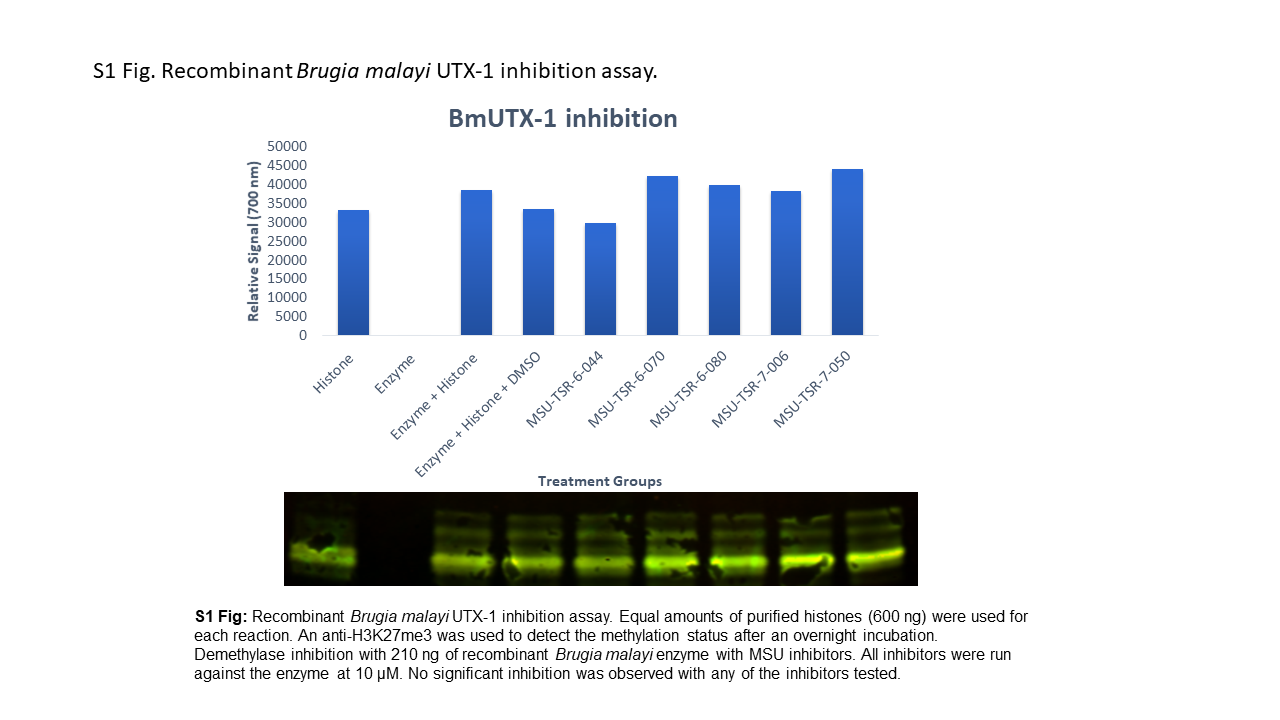

Supplement: S1 Fig — Tested compounds include MSU-TSR-038, 044, 104, 7–006, and GSK-J1. Based on the DMSO control lane, none of the compounds appeared to inhibit the ability for BmUTX-1 to demethylate the H3K27me3 mark. (TIF) [file pntd.0010216.s005.tif]
